# 3,4-Bis[4-(4-meth­oxy­phen­oxy)phen­yl]-2,5-dihydro­furan-2,5-dione

**DOI:** 10.1107/S1600536811015509

**Published:** 2011-05-07

**Authors:** Tao Zhang, Wenjing Wang, Jiao Xu, Liwei Ni, Xing Zhang

**Affiliations:** a College of Science, Northwest A&F University, Yangling 712100, Shannxi Province, People’s Republic of China; b Research and Development Center of Biorational Pesticide, Northwest A&F University, Yangling 712100, Shannxi Province, People’s Republic of China

## Abstract

In the crystal structure of the title compound, C_30_H_22_O_7_, neighbouring benzene rings are twisted out of the plane of the five-membered ring by 27.30 (3) and 45.47 (3)°.

## Related literature

For background to the use of 3,4-diaryl-substituted maleic anhydride derivatives as photochromic materials, see: Irie (2000 ▶). For related structures, see: Liu *et al.* (2003 ▶).
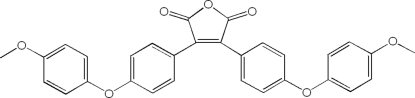


## Experimental

### 

#### Crystal data

C_30_H_22_O_7_
*M* *_r_* = 494.48
Orthorhombic, 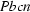

*a* = 16.981 (5) Å
*b* = 15.291 (5) Å
*c* = 18.665 (5) Å
*V* = 4846 (2) Å^3^
*Z* = 8
Mo *K*α radiation
μ = 0.10 mm^−1^
*T* = 293 K
0.32 × 0.30 × 0.26 mm

#### Data collection

Bruker APEXII CCD diffractometer
Absorption correction: multi-scan (*SADABS*; Sheldrick, 1998 ▶) *T*_min_ = 0.970, *T*_max_ = 0.975
21827 measured reflections
4522 independent reflections
2555 reflections with *I* > 2σ(*I*)
*R*_int_ = 0.059

#### Refinement

*R*[*F* ^2^ > 2σ(*F* ^2^)] = 0.044
*wR*(*F* ^2^) = 0.128
*S* = 1.10
4522 reflections
337 parameters
H-atom parameters constrained
Δρ_max_ = 0.23 e Å^−3^
Δρ_min_ = −0.21 e Å^−3^

### 

Data collection: *APEX2* (Bruker, 2005 ▶); cell refinement: *SAINT* (Bruker, 2005 ▶); data reduction: *SAINT*; program(s) used to solve structure: *SHELXS97* (Sheldrick, 2008 ▶); program(s) used to refine structure: *SHELXL97* (Sheldrick, 2008 ▶); molecular graphics: *SHELXTL* (Sheldrick, 2008 ▶); software used to prepare material for publication: *SHELXTL*.

## Supplementary Material

Crystal structure: contains datablocks global, I. DOI: 10.1107/S1600536811015509/nc2225sup1.cif

Structure factors: contains datablocks I. DOI: 10.1107/S1600536811015509/nc2225Isup2.hkl

Supplementary material file. DOI: 10.1107/S1600536811015509/nc2225Isup3.cml

Additional supplementary materials:  crystallographic information; 3D view; checkCIF report

## Figures and Tables

**Fig. 1.**
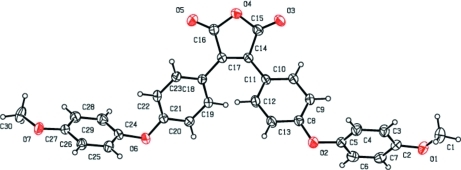
The molecular structure of (I). Displacement ellipsoids are drawn at the 30% probability level.

## supplementary crystallographic information

### Comment

3,4-Diaryl substituted maleic anhydride is a conjugated unit which has interesting optical and electronic properties. A number of 3,4-Diaryl substituted maleic anhydride derivatives have been designed and synthesized to be used as photochromic materials (Irie *et al.*, 2000; Liu *et al.*, 2003). In the course of exploring new photochromic compounds, we obtained an intermediate compound, which was characterized by single crystal X-ray analysis.

The molecule holds two long-chain branches with methyl group as the end to enhance its solubility. The interplanar angles between the two benzene rings connecting with the maleic anhydride five-membered ring are different. The interplanar angle between the benzene plane defined by C8, C9, C10, C11, C12, C13 and maleic anhydride plane is 27.30 (3) ° and that to the other benzene plane defined by C18, C19, C20, C21, C22, C23 amount to 45.47 (3) °.

### Experimental

3,4-bis(4-(4-methoxyphenoxy)phenyl)-*N*-methyl Maleimide(0.76 g, 1.5 mmol) and potassium hydroxide (0.67 g, 12 mmol) were dissolved in 60 ml of a 1: 1: 1 mixture of water, tetrahydrofuran and Methanol. The mixture was refluxed for 3 h and afterwards 3,4-bis(4(4-methoxyphenoxy)phenyl)maleic anhydride (0.69 g, 93%) was precipitated out by acidification with HCl. The product was dissolved in ethanol and yellow block crystals were formed on slow evaporation of the solvent at room temperature over one week.

### Crystal data

**Table:**

C_30_H_22_O_7_	*F*(000) = 2064
*M**_r_* = 494.48	*D*_x_ = 1.355 Mg m^−^^3^
Orthorhombic, *P**b**c**n*	Mo *K*α radiation, λ = 0.71069 Å
Hall symbol: -P 2n 2ab	Cell parameters from 3771 reflections
*a* = 16.981 (5) Å	θ = 2.4–22.4°
*b* = 15.291 (5) Å	µ = 0.10 mm^−^^1^
*c* = 18.665 (5) Å	*T* = 293 K
*V* = 4846 (2) Å^3^	Block, yellow
*Z* = 8	0.32 × 0.30 × 0.26 mm

### Data collection

**Table:**

Bruker APEXII CCD diffractometer	4522 independent reflections
Radiation source: fine-focus sealed tube	2555 reflections with *I* > 2σ(*I*)
graphite	*R*_int_ = 0.059
φ and ω scans	θ_max_ = 25.5°, θ_min_ = 2.1°
Absorption correction: multi-scan (*SADABS*; Sheldrick, 1998)	*h* = −20→19
*T*_min_ = 0.970, *T*_max_ = 0.975	*k* = −18→16
21827 measured reflections	*l* = −22→21

### Refinement

**Table:**

Refinement on *F*^2^	Secondary atom site location: difference Fourier map
Least-squares matrix: full	Hydrogen site location: inferred from neighbouring sites
*R*[*F*^2^ > 2σ(*F*^2^)] = 0.044	H-atom parameters constrained
*wR*(*F*^2^) = 0.128	*w* = 1/[σ^2^(*F*_o_^2^) + (0.0375*P*)^2^ + 2.1727*P*] where *P* = (*F*_o_^2^ + 2*F*_c_^2^)/3
*S* = 1.10	(Δ/σ)_max_ < 0.001
4522 reflections	Δρ_max_ = 0.23 e Å^−^^3^
337 parameters	Δρ_min_ = −0.21 e Å^−^^3^
0 restraints	Extinction correction: *SHELXL97* (Sheldrick, 2008), Fc^*^=kFc[1+0.001xFc^2^λ^3^/sin(2θ)]^-1/4^
Primary atom site location: structure-invariant direct methods	Extinction coefficient: 0.00128 (19)

### Special details

**Table:**

Geometry. All esds (except the esd in the dihedral angle between two l.s. planes) are estimated using the full covariance matrix. The cell esds are taken into account individually in the estimation of esds in distances, angles and torsion angles; correlations between esds in cell parameters are only used when they are defined by crystal symmetry. An approximate (isotropic) treatment of cell esds is used for estimating esds involving l.s. planes.
Refinement. Refinement of F^2^ against ALL reflections. The weighted R-factor wR and goodness of fit S are based on F^2^, conventional R-factors R are based on F, with F set to zero for negative F^2^. The threshold expression of F^2^ > 2sigma(F^2^) is used only for calculating R-factors(gt) etc. and is not relevant to the choice of reflections for refinement. R-factors based on F^2^ are statistically about twice as large as those based on F, and R- factors based on ALL data will be even larger.

### Fractional atomic coordinates and isotropic or equivalent isotropic displacement parameters (Å^2^)

**Table:**

	*x*	*y*	*z*	*U*_iso_*/*U*_eq_	
C1	0.10558 (15)	0.4872 (2)	0.51111 (18)	0.0764 (10)	
H1A	0.1019	0.4253	0.5193	0.115*	
H1B	0.0635	0.5054	0.4801	0.115*	
H1C	0.1016	0.5176	0.5560	0.115*	
C2	0.24659 (15)	0.49232 (17)	0.51718 (14)	0.0418 (6)	
C3	0.24878 (16)	0.45835 (19)	0.58530 (16)	0.0568 (8)	
H3	0.2023	0.4422	0.6080	0.068*	
C4	0.32052 (17)	0.44811 (19)	0.62036 (16)	0.0581 (8)	
H4	0.3221	0.4260	0.6668	0.070*	
C5	0.31599 (14)	0.51489 (18)	0.48274 (15)	0.0484 (7)	
H5	0.3145	0.5374	0.4365	0.058*	
C6	0.38727 (15)	0.50396 (18)	0.51708 (16)	0.0526 (7)	
H6	0.4339	0.5188	0.4939	0.063*	
C7	0.38916 (15)	0.47097 (17)	0.58578 (16)	0.0481 (7)	
C8	0.49774 (15)	0.38500 (17)	0.62627 (14)	0.0428 (6)	
C9	0.47156 (15)	0.31190 (18)	0.59006 (15)	0.0490 (7)	
H9	0.4278	0.3154	0.5602	0.059*	
C10	0.51081 (14)	0.23335 (17)	0.59853 (14)	0.0458 (7)	
H10	0.4924	0.1840	0.5747	0.055*	
C11	0.56429 (15)	0.37990 (17)	0.66916 (14)	0.0475 (7)	
H11	0.5822	0.4294	0.6931	0.057*	
C12	0.60384 (15)	0.30188 (17)	0.67634 (14)	0.0467 (7)	
H12	0.6489	0.2993	0.7046	0.056*	
C13	0.57726 (14)	0.22642 (16)	0.64184 (13)	0.0390 (6)	
C14	0.61543 (14)	0.14103 (16)	0.65403 (13)	0.0405 (6)	
C15	0.56624 (16)	0.06282 (18)	0.66906 (14)	0.0481 (7)	
C16	0.69217 (15)	0.11614 (16)	0.65787 (13)	0.0410 (6)	
C17	0.69275 (17)	0.02296 (18)	0.68064 (14)	0.0481 (7)	
C18	0.76504 (14)	0.16345 (16)	0.64095 (13)	0.0396 (6)	
C19	0.76455 (15)	0.23214 (19)	0.59134 (15)	0.0529 (8)	
H19	0.7175	0.2476	0.5693	0.063*	
C20	0.83223 (15)	0.27741 (19)	0.57450 (16)	0.0560 (8)	
H20	0.8305	0.3229	0.5415	0.067*	
C21	0.83686 (15)	0.14187 (18)	0.67179 (15)	0.0476 (7)	
H21	0.8391	0.0961	0.7045	0.057*	
C22	0.90562 (15)	0.18697 (18)	0.65504 (15)	0.0506 (7)	
H22	0.9531	0.1712	0.6763	0.061*	
C23	0.90295 (15)	0.25499 (17)	0.60682 (15)	0.0478 (7)	
C24	1.04147 (15)	0.28383 (19)	0.61559 (16)	0.0504 (7)	
C25	1.08571 (16)	0.2206 (2)	0.58285 (17)	0.0602 (8)	
H25	1.0652	0.1894	0.5444	0.072*	
C26	1.16158 (16)	0.20305 (19)	0.60732 (15)	0.0543 (8)	
H26	1.1917	0.1597	0.5855	0.065*	
C27	1.07113 (17)	0.33074 (19)	0.67234 (16)	0.0541 (8)	
H27	1.0408	0.3739	0.6940	0.065*	
C28	1.14612 (17)	0.31356 (18)	0.69705 (15)	0.0529 (7)	
H28	1.1660	0.3445	0.7359	0.063*	
C29	1.19195 (15)	0.25009 (17)	0.66392 (14)	0.0444 (7)	
C30	1.31494 (17)	0.1746 (2)	0.65874 (17)	0.0721 (10)	
H30A	1.2911	0.1181	0.6644	0.108*	
H30B	1.3657	0.1747	0.6814	0.108*	
H30C	1.3209	0.1873	0.6087	0.108*	
O1	0.17880 (9)	0.50672 (13)	0.47858 (10)	0.0559 (5)	
O2	0.46165 (10)	0.46629 (12)	0.62226 (11)	0.0620 (6)	
O3	0.49621 (12)	0.05362 (13)	0.66781 (11)	0.0656 (6)	
O4	0.61543 (11)	−0.00682 (12)	0.68610 (10)	0.0557 (5)	
O5	0.74626 (11)	−0.02544 (13)	0.69449 (11)	0.0614 (6)	
O6	0.96703 (10)	0.30546 (13)	0.58722 (12)	0.0648 (6)	
O7	1.26653 (10)	0.23887 (13)	0.69087 (10)	0.0587 (6)	

### Atomic displacement parameters (Å^2^)

**Table:**

	*U*^11^	*U*^22^	*U*^33^	*U*^12^	*U*^13^	*U*^23^
C1	0.0346 (16)	0.114 (3)	0.080 (2)	−0.0093 (18)	0.0067 (16)	0.001 (2)
C2	0.0356 (14)	0.0384 (16)	0.0515 (16)	0.0018 (12)	−0.0012 (13)	0.0010 (13)
C3	0.0433 (16)	0.0582 (19)	0.069 (2)	−0.0005 (14)	0.0059 (16)	0.0187 (16)
C4	0.0567 (19)	0.059 (2)	0.0584 (19)	0.0039 (16)	0.0007 (15)	0.0198 (15)
C5	0.0393 (15)	0.0589 (19)	0.0470 (16)	0.0016 (14)	0.0039 (13)	0.0060 (14)
C6	0.0362 (15)	0.0557 (19)	0.066 (2)	0.0027 (14)	0.0097 (14)	0.0023 (15)
C7	0.0404 (16)	0.0405 (16)	0.0632 (19)	0.0102 (13)	−0.0078 (14)	0.0023 (14)
C8	0.0369 (15)	0.0401 (16)	0.0512 (17)	0.0034 (12)	−0.0015 (12)	0.0054 (13)
C9	0.0358 (15)	0.0537 (18)	0.0577 (18)	0.0045 (14)	−0.0107 (13)	0.0022 (15)
C10	0.0396 (15)	0.0450 (16)	0.0527 (17)	−0.0006 (13)	−0.0060 (13)	−0.0030 (13)
C11	0.0464 (16)	0.0408 (17)	0.0553 (18)	−0.0025 (13)	−0.0113 (14)	−0.0016 (14)
C12	0.0374 (15)	0.0470 (17)	0.0556 (18)	0.0026 (13)	−0.0096 (13)	0.0040 (14)
C13	0.0328 (14)	0.0410 (16)	0.0432 (15)	0.0012 (12)	−0.0005 (11)	0.0023 (12)
C14	0.0388 (15)	0.0393 (15)	0.0434 (15)	0.0033 (12)	−0.0017 (12)	0.0024 (12)
C15	0.0427 (17)	0.0508 (18)	0.0508 (17)	0.0025 (15)	−0.0017 (14)	0.0040 (14)
C16	0.0408 (15)	0.0381 (15)	0.0441 (15)	0.0039 (12)	−0.0017 (12)	0.0053 (12)
C17	0.0487 (17)	0.0472 (18)	0.0485 (17)	0.0045 (15)	0.0018 (14)	0.0064 (14)
C18	0.0380 (15)	0.0381 (15)	0.0428 (15)	0.0075 (12)	0.0009 (12)	0.0026 (12)
C19	0.0378 (16)	0.060 (2)	0.0606 (18)	0.0079 (14)	−0.0007 (13)	0.0196 (16)
C20	0.0450 (17)	0.0570 (19)	0.0659 (19)	0.0076 (14)	0.0028 (14)	0.0242 (15)
C21	0.0425 (16)	0.0444 (16)	0.0558 (17)	0.0041 (13)	−0.0034 (14)	0.0096 (14)
C22	0.0382 (15)	0.0515 (18)	0.0622 (19)	0.0048 (13)	−0.0066 (14)	0.0081 (15)
C23	0.0389 (15)	0.0448 (17)	0.0599 (18)	0.0051 (13)	0.0050 (13)	0.0061 (14)
C24	0.0365 (15)	0.0466 (18)	0.068 (2)	−0.0019 (14)	0.0012 (14)	0.0120 (16)
C25	0.0524 (18)	0.0584 (19)	0.070 (2)	0.0029 (16)	−0.0142 (16)	−0.0164 (17)
C26	0.0471 (17)	0.0562 (19)	0.0597 (19)	0.0074 (14)	−0.0067 (14)	−0.0150 (15)
C27	0.0547 (19)	0.0475 (18)	0.0600 (19)	0.0018 (15)	0.0183 (15)	0.0011 (15)
C28	0.0583 (19)	0.0524 (18)	0.0479 (17)	−0.0093 (15)	0.0055 (14)	−0.0051 (14)
C29	0.0410 (15)	0.0485 (17)	0.0438 (15)	−0.0068 (13)	0.0006 (13)	−0.0016 (13)
C30	0.0463 (18)	0.100 (3)	0.070 (2)	0.0106 (18)	−0.0022 (16)	−0.007 (2)
O1	0.0332 (10)	0.0699 (14)	0.0645 (13)	−0.0002 (10)	−0.0018 (9)	0.0077 (10)
O2	0.0480 (12)	0.0471 (12)	0.0908 (16)	0.0059 (9)	−0.0257 (11)	0.0052 (11)
O3	0.0442 (12)	0.0672 (14)	0.0855 (15)	−0.0093 (11)	−0.0017 (11)	0.0142 (12)
O4	0.0503 (12)	0.0458 (12)	0.0710 (14)	0.0002 (10)	0.0047 (10)	0.0139 (10)
O5	0.0563 (12)	0.0495 (12)	0.0785 (14)	0.0119 (10)	0.0023 (11)	0.0183 (11)
O6	0.0409 (11)	0.0603 (13)	0.0933 (16)	−0.0012 (10)	0.0009 (11)	0.0254 (12)
O7	0.0444 (12)	0.0734 (14)	0.0583 (12)	−0.0048 (10)	−0.0091 (9)	−0.0092 (11)

### Geometric parameters (Å, °)

**Table:**

C1—O1	1.416 (3)	C16—C18	1.468 (3)
C1—H1A	0.9600	C16—C17	1.487 (4)
C1—H1B	0.9600	C17—O5	1.200 (3)
C1—H1C	0.9600	C17—O4	1.393 (3)
C2—C3	1.374 (4)	C18—C21	1.388 (3)
C2—O1	1.376 (3)	C18—C19	1.400 (3)
C2—C5	1.386 (3)	C19—C20	1.378 (4)
C3—C4	1.392 (4)	C19—H19	0.9300
C3—H3	0.9300	C20—C23	1.387 (4)
C4—C7	1.377 (4)	C20—H20	0.9300
C4—H4	0.9300	C21—C22	1.392 (3)
C5—C6	1.380 (3)	C21—H21	0.9300
C5—H5	0.9300	C22—C23	1.376 (4)
C6—C7	1.378 (4)	C22—H22	0.9300
C6—H6	0.9300	C23—O6	1.383 (3)
C7—O2	1.408 (3)	C24—C25	1.369 (4)
C8—C9	1.380 (4)	C24—C27	1.375 (4)
C8—C11	1.387 (3)	C24—O6	1.410 (3)
C8—O2	1.388 (3)	C25—C26	1.393 (4)
C9—C10	1.383 (3)	C25—H25	0.9300
C9—H9	0.9300	C26—C29	1.378 (4)
C10—C13	1.392 (3)	C26—H26	0.9300
C10—H10	0.9300	C27—C28	1.380 (4)
C11—C12	1.376 (3)	C27—H27	0.9300
C11—H11	0.9300	C28—C29	1.389 (4)
C12—C13	1.396 (4)	C28—H28	0.9300
C12—H12	0.9300	C29—O7	1.373 (3)
C13—C14	1.475 (3)	C30—O7	1.415 (3)
C14—C16	1.360 (3)	C30—H30A	0.9600
C14—C15	1.486 (4)	C30—H30B	0.9600
C15—O3	1.198 (3)	C30—H30C	0.9600
C15—O4	1.390 (3)		
			
O1—C1—H1A	109.5	O5—C17—O4	119.7 (2)
O1—C1—H1B	109.5	O5—C17—C16	131.1 (3)
H1A—C1—H1B	109.5	O4—C17—C16	109.1 (2)
O1—C1—H1C	109.5	C21—C18—C19	117.2 (2)
H1A—C1—H1C	109.5	C21—C18—C16	122.3 (2)
H1B—C1—H1C	109.5	C19—C18—C16	120.5 (2)
C3—C2—O1	124.6 (2)	C20—C19—C18	121.5 (2)
C3—C2—C5	120.0 (2)	C20—C19—H19	119.2
O1—C2—C5	115.4 (2)	C18—C19—H19	119.2
C2—C3—C4	120.1 (3)	C19—C20—C23	119.9 (3)
C2—C3—H3	120.0	C19—C20—H20	120.0
C4—C3—H3	120.0	C23—C20—H20	120.0
C7—C4—C3	119.5 (3)	C18—C21—C22	121.7 (2)
C7—C4—H4	120.3	C18—C21—H21	119.1
C3—C4—H4	120.3	C22—C21—H21	119.1
C6—C5—C2	120.0 (3)	C23—C22—C21	119.6 (2)
C6—C5—H5	120.0	C23—C22—H22	120.2
C2—C5—H5	120.0	C21—C22—H22	120.2
C7—C6—C5	119.8 (3)	C22—C23—O6	124.7 (2)
C7—C6—H6	120.1	C22—C23—C20	120.0 (3)
C5—C6—H6	120.1	O6—C23—C20	115.4 (2)
C4—C7—C6	120.6 (3)	C25—C24—C27	120.8 (3)
C4—C7—O2	120.0 (3)	C25—C24—O6	119.4 (3)
C6—C7—O2	119.3 (2)	C27—C24—O6	119.7 (3)
C9—C8—C11	120.0 (2)	C24—C25—C26	119.8 (3)
C9—C8—O2	123.8 (2)	C24—C25—H25	120.1
C11—C8—O2	116.2 (2)	C26—C25—H25	120.1
C8—C9—C10	119.5 (2)	C29—C26—C25	119.8 (3)
C8—C9—H9	120.3	C29—C26—H26	120.1
C10—C9—H9	120.3	C25—C26—H26	120.1
C9—C10—C13	121.5 (2)	C24—C27—C28	119.8 (3)
C9—C10—H10	119.2	C24—C27—H27	120.1
C13—C10—H10	119.2	C28—C27—H27	120.1
C12—C11—C8	120.2 (2)	C27—C28—C29	120.1 (3)
C12—C11—H11	119.9	C27—C28—H28	120.0
C8—C11—H11	119.9	C29—C28—H28	120.0
C11—C12—C13	120.9 (2)	O7—C29—C26	124.1 (2)
C11—C12—H12	119.5	O7—C29—C28	116.2 (2)
C13—C12—H12	119.5	C26—C29—C28	119.7 (3)
C10—C13—C12	117.8 (2)	O7—C30—H30A	109.5
C10—C13—C14	120.9 (2)	O7—C30—H30B	109.5
C12—C13—C14	121.2 (2)	H30A—C30—H30B	109.5
C16—C14—C13	132.6 (2)	O7—C30—H30C	109.5
C16—C14—C15	107.7 (2)	H30A—C30—H30C	109.5
C13—C14—C15	119.6 (2)	H30B—C30—H30C	109.5
O3—C15—O4	120.7 (3)	C2—O1—C1	118.5 (2)
O3—C15—C14	130.5 (3)	C8—O2—C7	117.2 (2)
O4—C15—C14	108.8 (2)	C15—O4—C17	107.4 (2)
C14—C16—C18	131.2 (2)	C23—O6—C24	118.4 (2)
C14—C16—C17	106.8 (2)	C29—O7—C30	117.9 (2)
C18—C16—C17	121.9 (2)		
